# A phase II open label, single arm study of hypofractionated stereotactic radiotherapy with chemoradiotherapy using intensity-modulated radiotherapy for newly diagnosed glioblastoma after surgery: the HSCK-010 trial protocol

**DOI:** 10.1186/s12885-022-09914-5

**Published:** 2022-07-29

**Authors:** Yun Guan, Mingyuan Pan, Jun Yang, Qiuxia Lu, Liangfu Han, Ying Liu, Jing Li, Huaguang Zhu, Xiu Gong, Guanghai Mei, Xiaoxia Liu, Li Pan, Jiazhong Dai, Yang Wang, Enmin Wang, Xin Wang

**Affiliations:** 1 CyberKnife Center, Department of Neurosurgery, Huashan Hospital, Shanghai Medical College, Fudan University, 12 Wulumuqi Road (M), Shanghai, 200040 China; 2 National Center for Neurological Disorders, Shanghai, China; 3 Shanghai Key Laboratory of Brain Function and Restoration and Neural Regeneration, Shanghai, China; 4 Neurosurgical Institute of Fudan University, Shanghai, China; 5 Shanghai Clinical Medical Center of Neurosurgery, Shanghai, China; 6 Foshan Chancheng Hospital, Foshan, China; 7 Department of Pathology, School of Basic Medical Sciences, Fudan University, Shanghai, China

**Keywords:** Hypofractionated stereotactic radiotherapy, Newly diagnosed glioblastoma, Adjuvant chemoradiotherapy

## Abstract

**Background:**

The most frequently diagnosed primary brain tumor is glioblastoma (GBM). Nearly all patients experience tumor recurrence and up to 90% of which is local recurrence. Thus, increasing the therapeutic ratio of radiotherapy using hypofractionated stereotactic radiotherapy (HSRT) can reduce treatment time and may increase tumor control and improve survival. To evaluate the efficacy and toxicity of the combination of HSRT and intensity-modulated radiotherapy (IMRT) with temozolomide after surgery in GBM patients and provide evidence for further randomized controlled trials.

**Methods/design:**

HSCK-010 is an open-label, single-arm phase II trial (NCT04547621) which includes newly diagnosed GBM patients who underwent gross total resection. Patients will receive the combination of 30 Gy/5fx HSRT, and 20 Gy/10fx IMRT adjuvant therapy with concurrent temozolomide and adjuvant chemotherapy. The primary endpoint is overall survival (OS). Secondary outcomes include progression-free survival (PFS) rate, objective-response rate (ORR), quality of life (Qol) before and after the treatment, cognitive function before and after the treatment, and rate of treatment-related adverse events (AE). The combination of HSRT and IMRT with temozolomide can benefit the patients after surgery with good survival, acceptable toxicity, and reduced treatment time.

**Trial registration:**

NCT04547621. Registered on 14 September 2020.

## Background

The most frequently diagnosed primary brain tumor is glioblastoma (GBM). In the United States [1], there are 2.96 newly diagnosed occurrences per 100,000 people per year while the number in China is 5 to 8. Despite definitive primary therapy including surgery, adjuvant 60 Gy/30fx chemoradiation, and temozolomide based chemotherapy [2], nearly all patients experience tumor recurrence [3] and up to 90% of which is local recurrence [4]. Thus, increasing the therapeutic ratio of radiotherapy may result in better tumor control and improve overall survival (OS).

Hypofractionated stereotactic radiotherapy (HSRT) can increase the therapeutic ratio by increasing the effectiveness and decreasing the side effects. Also, the treatment can be delivered in a shorter duration compared with conventional radiotherapy (RT). Several trials have evaluated the hypothesis of using HSRT in newly diagnosed glioblastoma multiforme (GBM) patients. Roa, et al. reported the 40 Gy/15fx HSRT improved OS compared with 60 Gy/30fx conventional RT (5.6 months vs 5.1 months, *P* < 0.05) in a randomized trial [5]. Roa et al. also reported the OS of 7.9 months using 25 Gy/5fx HSRT in elderly and/or frail GMB patients later in 2015[6]. Another randomized phase 3 trial compared temozolomide, 60 Gy/30fx standard RT and 34 Gy/10fx HSRT. For patients older than 70, the survival was better with HSRT than with standard RT (HR 0.59, 95%CI 0.37 – 0.93, *p* = 0.02). HSRT resulted in lower toxicity and shorter treatment duration.

The RTOG 93–05 reported a negative result of comparing conventional RT with carmustine alone or followed by radiosurgery [6]. To date, limited data were reported about the effectiveness of HSRT combined with intensity-modulated radiotherapy (IMRT) as adjuvant therapy in newly diagnosed GBM. With the development of HSRT techniques, there is a clear rationale for combing IMRT with HSRT to increase the treatment ratio and reduce active treatment time.

**Fig. 1:**
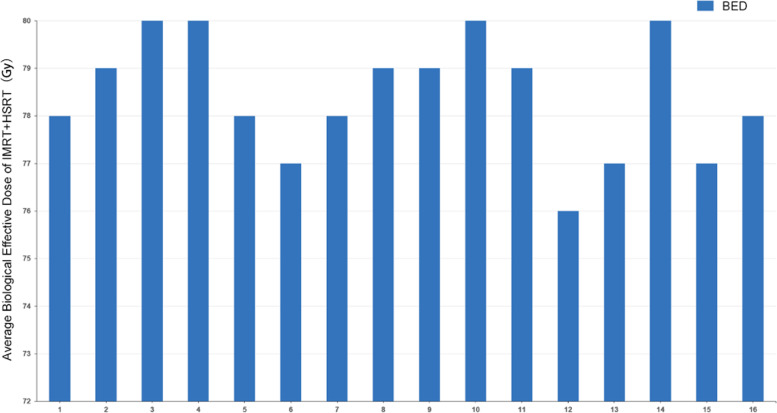
The average biological effective dose calculated using the formula BED = Total dose * (1 + (Fraction dose /α/β)), α/β ratio = 10. Silico study of 16 newly diagnosed glioblastoma patients resulted a higher BED compared with the conventional radiotherapy regimen. BED: biological effective dose; IMRT: Intensity-modulated radiation therapy; HSRT: Hypofractionated stereotactic radiotherapy

New generation automated noncoplanar HSRT delivery systems can deliver high-dose treatment and limit the dose to normal structures. However, few studies have examined the role of this new technique in newly diagnosed GBM. This study aims to evaluate the safety and effectiveness of the combination of 30 Gy/5fx HSRT and 20 Gy/10fx IMRT adjuvant therapy. The average total biological effective dose (BED) of the regimen is higher than the conventional 60 Gy/30fx treatment in our previous silico study (Fig. 1). This study can provide evidence for future non-inferiority phase III randomized controlled trials. The abbreviated course of radiotherapy can reduce the treatment time by half, benefit patients, and utilize the health resource. Thus, this study is important in potentially changing the paradigm of newly diagnosed GBM treatment.

## Method/design

### Hypothesis

The hypothesis is that the combination of HSRT and IMRT with temozolomide which has the same BED compared with the conventional 60 Gy/30fx. In our previous silico study, the average BED delivered to the gross tumor volume (GTV) is higher than the conventional regimen (Fig. 1). The treatment can benefit the patients after surgery with similar efficacy and reduced treatment time. Thus, the goal is to evaluate the efficacy and toxicity of this regimen and provide evidence for further randomized controlled trials.

### Primary outcome

The primary endpoint of this open-label, single-arm phase II trial is the overall survival in GBM patients receiving hypofractionated stereotactic radiotherapy plus chemoradiotherapy after surgery.

### Secondary outcomes

To estimate the following factors:

- The progression-free survival (PFS) rate.
- The objective-response rate (ORR).
- The quality of life (Qol) before and after the treatment.
- The cognitive function before and after the treatment.
- The rate of treatment-related adverse events (AE).

## Study design

### Inclusion criteria

- 18–70 years of age;
- Karnofsky performance status (KPS) ≥ 60 within 14 days prior to registration;
- Histopathologically proved diagnosis glioblastoma multiforme;
- Underwent surgery, gross total resection, or subtotal resection;
- Estimated survival of at least 3 months;
- Hgb > 90/gL; absolute neutrophil count (ANC) > 1.5 × 109/L, platelets > 80 × 109/L; Creatinine < 1.5 times the upper limit of laboratory normal value; Bilirubin < 2 times the upper limit of laboratory normal value; serum glutamate pyruvate transaminase (SGPT) or serum glutamate oxaloacetate transaminase (SGOT) < 3 times the upper limit of laboratory normal value;
- Signed informed consent form;
- Agreed to participate in the follow-up.

### Exclusion criteria

- Prior invasive malignancy unless disease-free;
- Received irradiation or other anti-tumor adjuvant therapies;
- Brain stem disease or tumor greater than 6 cm in maximum diameter;
- Isocitrate dehydrogenase (IDH) 1 or IDH2 mutations;
- Prior therapy with an inhibitor of vascular endothelial growth factor (VEGF) or VEGFR;
- Pregnancy or nursing mothers;
- Participated in other trials after diagnosis;
- Influence factors toward oral medications;
- Patients with Common Terminology Criteria for Adverse Events (CTCAE) 5.0 grade 3 + bleeding within 4 weeks prior to registration;
- Suffering from severe cardiovascular disease: myocardial ischemia or myocardial infarction above grade II, poorly controlled arrhythmias (including men with QTc interval ≥ 450 ms, women ≥ 470 ms); according to NYHA criteria, grades III to IV Insufficient function, or cardiac color Doppler ultrasound examination indicates left ventricular ejection fraction (LVEF) < 50%;
- Long-term unhealed wounds or fractures;
- History of organ transplantation;
- Serious diseases that endanger patients’ safety or affect patients’ completion of research, according to the researchers’ judgment.

### Withdrawal criteria

Subjects may discontinue participating in the study including but not limited to the following reasons:

- Progressive disease according to RANO criteria [7];
- Intolerable treatment-related toxicity;
- Protocol violation;
- Patient or investigator’s request;
- Non-compliance of the patient;
- Trial termination or death.

## Methodology

### Treatments

The regimen will be 3 major parts.

- IMRT radiotherapy;
- HSRT radiotherapy;
- Temozolomide systemic treatment.

IMRT is recommended to be administered within 3 to 5 weeks after the surgery. A magnetic resonance imaging (MRI) and computerized tomography (CT) scanning obtained with the patient immobilized in the treatment is required for treatment planning. The gross tumor volume (GTV) will be defined by T1 enhancement and either the FLAIR or T2 abnormality on the postoperative MRI scan. The clinical target volume (CTV) will be defined by the GTV plus a margin of 2 cm to include the tumor subregion. The planning tumor volume (PTV) will be defined by the CTV plus a margin of 3 to 5 mm to account for the set-up and localization. It can be acceptable to reduce the PTV margin if the organ at risk (OAR) is not permissible. The PTV will be treated to 20 Gy in 10 fractions, once daily. The IMRT treatment will be finished within 2 weeks. The acceptable plan will be defined as >  = 90% of PTV covered by 20 Gy and >  = 97% of PTV covered by 18 Gy. The unacceptable plan will be defined as < 90% of PTV covered by 20 Gy and < 97% of PTV covered by 18 Gy.

The HSRT treatment should be administered within 3 days after the IMRT. The GTV will be defined by the contrast-enhanced MRI T1 scan and the surgical cavity margins. The CTV will be defined by the GTV plus a margin of 5 mm, and the PTV will be the same as the CTV. The HSRT treatment should be administered within 3 days after the IMRT. The PTV will be treated to 30 Gy in 5 fractions once daily, and an iso-dose line of 60–70% will be acceptable. The radiotherapy combination will be 30 Gy/5fx HSRT and 20 Gy/10fx IMRT. The total biological effective dose (BED) of the PTV is 72 Gy in a ratio of alpha/beta ratio of 10, which equals the conventional 60 Gy/30fx treatment but halved the duration.

**Fig. 2:**
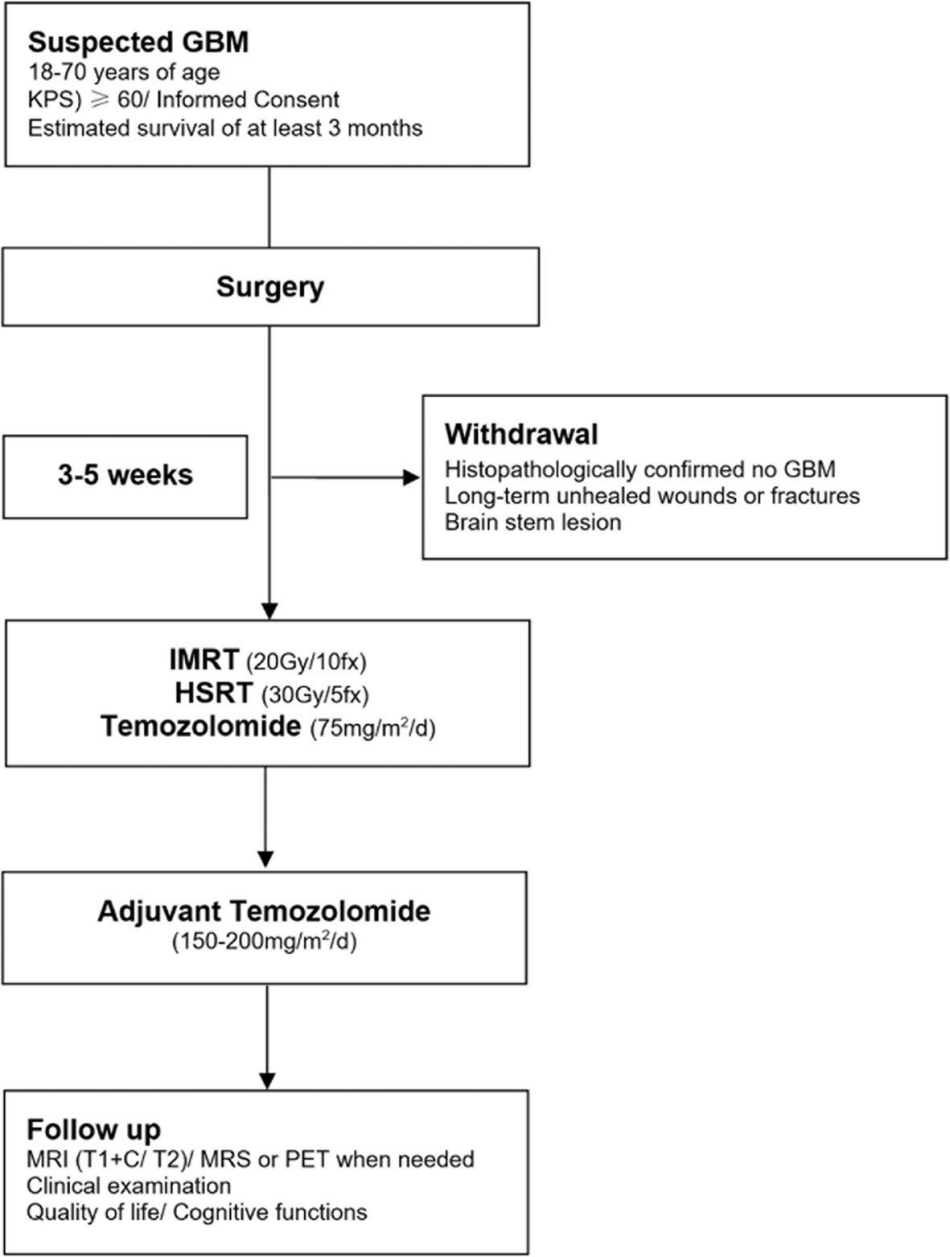
The workflow of the study. GBM: Glioblastoma multiforme; IMRT: Intensity-modulated radiation therapy; HSRT: Hypofractionated stereotactic radiotherapy; MRI: magnetic resonance imaging; MRS: magnetic resonance spectroscopy; PET: positron emission tomography

Temozolomide will be administered on the first day of IMRT concurrently till the last day of HSRT at a daily oral dose of 75 mg/m^2^. Temozolomide is recommended to be taken in the morning. Post-radiation temozolomide will be administered 28 days after the last day of radiotherapy. A variation of 3 days will be acceptable. The first cycle will be 150 mg/m^2^ daily for 5 days of a 28-day cycle. If no > grade 2 toxicity is observed. The following treatment will be temozolomide 200 mg/m^2^ for at least 6 cycles (Fig. 2).

### Sample size and power justification

The design will be a prospective single-arm phase II trial to determine the effectiveness and safety of HSRT plus chemoradiotherapy after surgery for GBM. A two-sided, one-sample log-rank test calculated from a sample of 45 subjects achieves 80% power at a 0.050 significance level to detect a hazard ratio of 0.580 when the median survival time of the historic control group is 14 months. Subjects are accrued for 12 months. Follow-up continues for 24 months after the last subject is added. Assuming a 10% loss to follow-up, the actual sample size will be 50.

### Statistical methods

The final analysis will be done on an intent-to-treat group. The median and 95% Confidence intervals (CIs) of OS and PFS will be estimated using the Kaplan–Meier method. Overall survival will be measured from the date of treatment to the date of death or the last follow-up. Progression-free survival will be measured from the date of treatment to the date of first progression or death or the last follow-up.

ORR will be based on the proportion of patients with a best overall response of confirmed CR or PR. ORR 95% will be estimated using the exact binomial method based on the F-distribution.

EORTC QLQ-C30 (version 3.0) questionnaire will be used to evaluate the quality of life. All scales range in score from 0 to 100. The mean scores will be calculated at baseline and every 2 months after the treatment. The mean ± SD, maximum, minimum, and median values will be used to describe the measurement before and after the treatment.

Mini-Mental State Exam (MMSE, score range 0 to 30) will be used to evaluate the cognitive function. Any score of 24 or more (out of 30) indicates normal cognition. Below this, scores can indicate severe (≤ 9 points), moderate (10–18 points), or mild (19–23 points) cognitive impairment.

The mean ± SD, maximum, minimum, and median values will be used to describe the measurement before and after the treatment.

CTCAE 5.0 will be used to assess the toxicity. The number of events, number of subjects, and the incidence rate will be used to describe the measurement.

### Evaluation, neurological, and quality of life

The assessment will be done every two months, which will include the medical history, vital signs, physical examination, KPS, blood pressure, EKG, MRI (T1 Gad enhanced, T2 FLAIR), blood routine, blood biochemistry, urine routine, stool routine, quality of life score (QLQ-C30 3.0) and cognitive evaluation (MMSE). The measure of response will be by the RANO criteria. An overview of the study assessments and procedures is presented in Table 1.

**Table 1:** Data collection schedule

Follow-up							
	Inclusion	Surgery	RT	M2	M4	M6	Mx^a^
Consent	✓						
Medical History	✓						
Physical examinations and vital signs	✓			✓	✓	✓	✓
Blood routine, Hepatic and Renal Function	✓	✓	✓	✓	✓	✓	✓
Pregnancy Test (Childbearing Age Women)	✓						
Dosimetric MRI + CT Scan			✓				
MRI (T1 enhanced, T2 FLAIR)		✓		✓	✓	✓	✓
KPS Score	✓	✓	✓	✓	✓	✓	✓
QLQ-C30 questionnaire	✓	✓	✓	✓	✓	✓	✓
MMSE questionnaire	✓	✓	✓	✓	✓	✓	✓
Toxicity Evaluation (CTCAE 5.0)	✓	✓	✓	✓	✓	✓	✓

^a^The follow-up will be done every two months till death or the patient withdrawal from the study

### Data management

All data will be recorded in the CRF and submitted to the data center. The study investigators will collect and restore pseudonymized electronically in compliance with local regulations. Pseudonymized data will be accessible to all investigators and full data access will be given to related supervising authorities.

### Quality assurance and safety considerations

The treatment-related adverse events will be defined according to the CTCAE 5.0. The AE will be monitored and recorded by laboratory tests, clinical examinations, and MRI. Acute neurotoxicity will be defined as any treatment-related neurologic adverse events occurring within 4 weeks after the last day of radiotherapy. Late neurotoxicity will be classified as those that happen 3 months after radiotherapy, and the rest (4 weeks to 3 months) defined as early delayed neurotoxicity. Pseudoprogression and radiation necrosis (RN) are common events in brain tumor patients who underwent radiotherapy [8]. MR Spectroscopy (MRS) or L-[methyl-(11)C]-methionine (MET) positron emission tomography (PET) will be considered to distinguish the events [9, 10].

The Institutional Review Board (IRB) will receive the report of all recorded adverse events exceeding CTCAE 5.0 Grade 2 for evaluation. Any serious adverse events will be reported to the IRB within 24 h.

### Project duration and expected outcomes

The estimated period of inclusion time will be 1 year. The estimated study completion date will be the end of 2021. And the follow-up duration will be 2 years. The complete duration is estimated to be 3 years.

The study is expected to have a similar PFS, and OS compared with the survival of standard chemoradiation reported by Stupp. If the result supports the hypothesis, a non-inferiority phase III randomized controlled trial will be conducted to provide higher evidence to support this regimen. The abbreviated course of radiotherapy can reduce the treatment time by half, benefit patients, and utilize the health resource.

## Discussion

GBM is the most common malignant primary brain tumor, and it has a poor prognosis despite the definite standard treatment of surgery and chemoradiotherapy. New generation automated noncoplanar HSRT delivery systems can deliver high-dose treatment and limit the dose to normal structures. The hypofractionated radiotherapy has been reported to have a similar OS compared with the standard 60 Gy dose radiotherapy and is considered to be the standard care for elder patients [11, 12].

This trial aims at assessing the safety and effec1tiveness of the combination of 30 Gy/5fx HSRT using 20 Gy/10fx IMRT as adjuvant treatment for newly diagnosed GBM patients. The radiotherapy regimen has the same BED compared to the conventional dose and can reduce the treatment time by half which can benefit the patients, especially in the case of GBM which has a relatively short life expectancy. The follow-up imaging information, quality of life, and cognitive functions will also be analyzed to evaluate the regimen. Also, the results may provide evidence for phase III randomized controlled trials to further investigate the best short radiotherapy schemes.

## Data Availability

Not applicable.

## Abbreviations

GBM: Glioblastoma
PTV: Planning target volume
KPS: Karnofsky Performance Status
OS: Overall survival
PFS: Progression-free survival
WHO: World Health Organization
IDH: Isocitrate dehydrogenase
GTV: Gross tumor volume
CTV: Clinical tumor volume
BED: Biologically effective dose
LR: Local recurrence
HSRS: Hypofractionated stereotactic radiosurgery
RCT: Randomized control trials
HSRT: Hypofractionated stereotactic radiotherapy

## References

[1] Jemal A, Siegel R, Ward E (2006). Cancer statistics. CA Cancer J Clin.

[2] Stupp R, Mason WP, van den Bent MJ (2005). Radiotherapy plus concomitant and adjuvant temozolomide for glioblastoma. N Engl J Med.

[3] Kirkpatrick JP, Sampson JH (2014). Recurrent malignant gliomas. Semin Radiat Oncol.

[4] Martínez-Carrillo M, Tovar-Martín I, Zurita- Herrera M (2014). Salvage radiosurgery for selected patients with recurrent malignant gliomas. Biomed Res Int.

[5] Roa W, Brasher PMA, Bauman G (2004). Abbreviated course of radiation therapy in older patients with glioblastoma multiforme: a prospective randomized clinical trial. J Clin Oncol.

[6] Roa W, Kepka L, Kumar N (2015). International Atomic Energy Agency randomized phase III study of radiation therapy in elderly and/or frail patients with newly diagnosed glioblastoma multiforme. J Clin Oncol.

[7] Vogelbaum MA, Jost S, Aghi MK (2012). Application ofnovel response/progression measures for surgically delivered therapies for gliomas: response assessment in neuro-oncology (RANO) working group. Neurosurgery.

[8] Brandsma D (2008). Clinical features, mechanisms, and management of pseudoprogression in malignant gliomas. Lancet Oncol.

[9] Suh CH, Kim HS, Choi YJ (2013). Prediction of pseudoprogression in patients with glioblastomas using the initial and final area under the curve’s ratio derived from dynamic contrast-enhanced T1-weighted perfusion MR imaging. AJNR Am JNeuroradiol.

[10] Guan Y (2020). Hypofractionated Radiosurgery Plus Bevacizumab for Locally Recurrent Brain Metastasis with Previously High-Dose Irradiation. World Neurosurg.

[11] Malmström A (2012). Temozolomide versus standard 6-week radiotherapy versus hypofractionated radiotherapy in patients older than 60 years with glioblastoma: the Nordic randomised, phase 3 trial. Lancet Oncol.

[12] Perry JR (2017). Short-course radiation plus temozolomide in elderly patients with glioblastoma. N Engl J Med.

